# Supplementary material for: Trips and neurotransmitters: Discovering principled patterns across 6850 hallucinogenic experiences
Source: Sci Adv. 2022 Mar 16;8(11):eabl6989. doi: 10.1126/sciadv.abl6989 (PMC8926331; doi:10.1126/sciadv.abl6989)
Supplement: Supplementary file 1 — Figs. S1 to S7 Table S2 [file sciadv.abl6989_sm.pdf]

Science Advances
AAAS

Supplementary Materials for

Trips and neurotransmitters: Discovering principled patterns across 6850 hallucinogenic experiences

Galen Ballentine, Samuel Freesun Friedman, Danilo Bzdok*

*Corresponding author. Email: danilo.bzdok@mcgill.ca

Published 16 March 2022, *Sci. Adv.* **8**, eabl6989 (2022)
DOI: 10.1126/sciadv.abl6989

The PDF file includes:

Figs. S1 to S7
Table S2

Other Supplementary Material for this manuscript includes the following:

Table S1

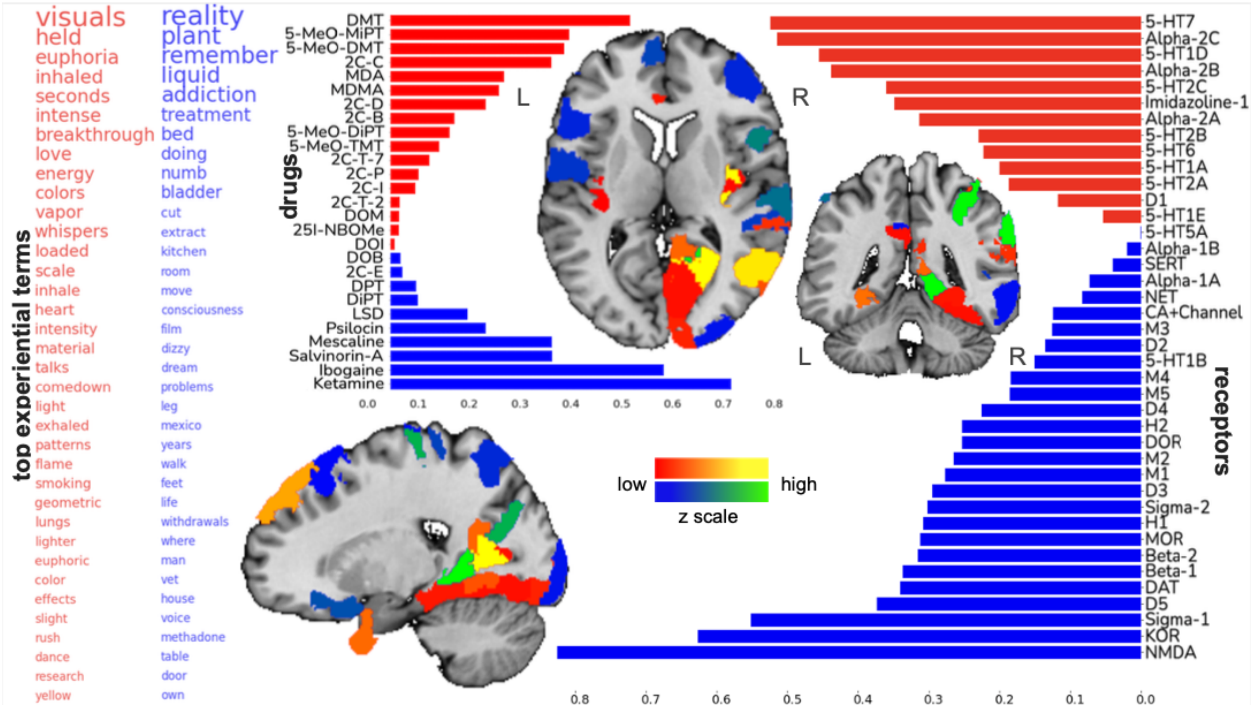

Supplementary Figure 1: The fourth factor underlying hallucinogenic experiences. Sagittal, coronal, and axial brain slices are shown at x=20, y=-43, and z=8 (MNI reference space).

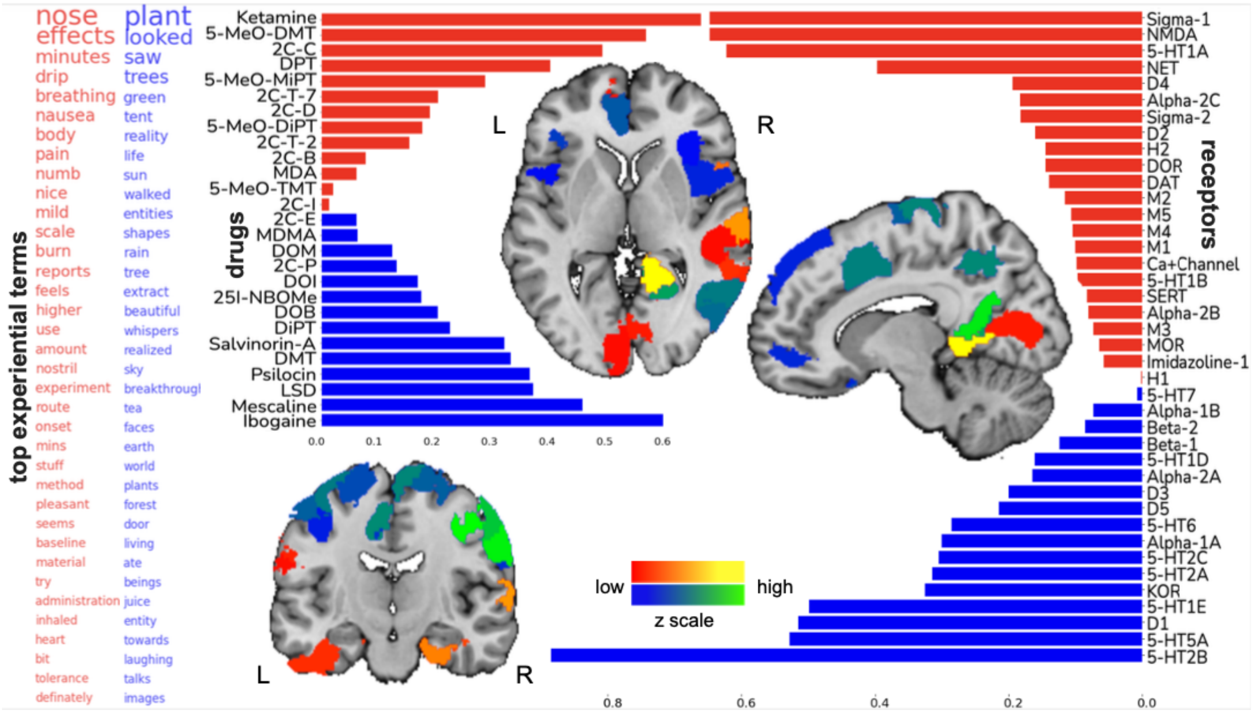

Supplementary Figure 2: The fifth factor underlying hallucinogenic experiences. Sagittal, coronal, and axial brain slices are shown at x=11, y=-15, and z=2 (MNI reference space).

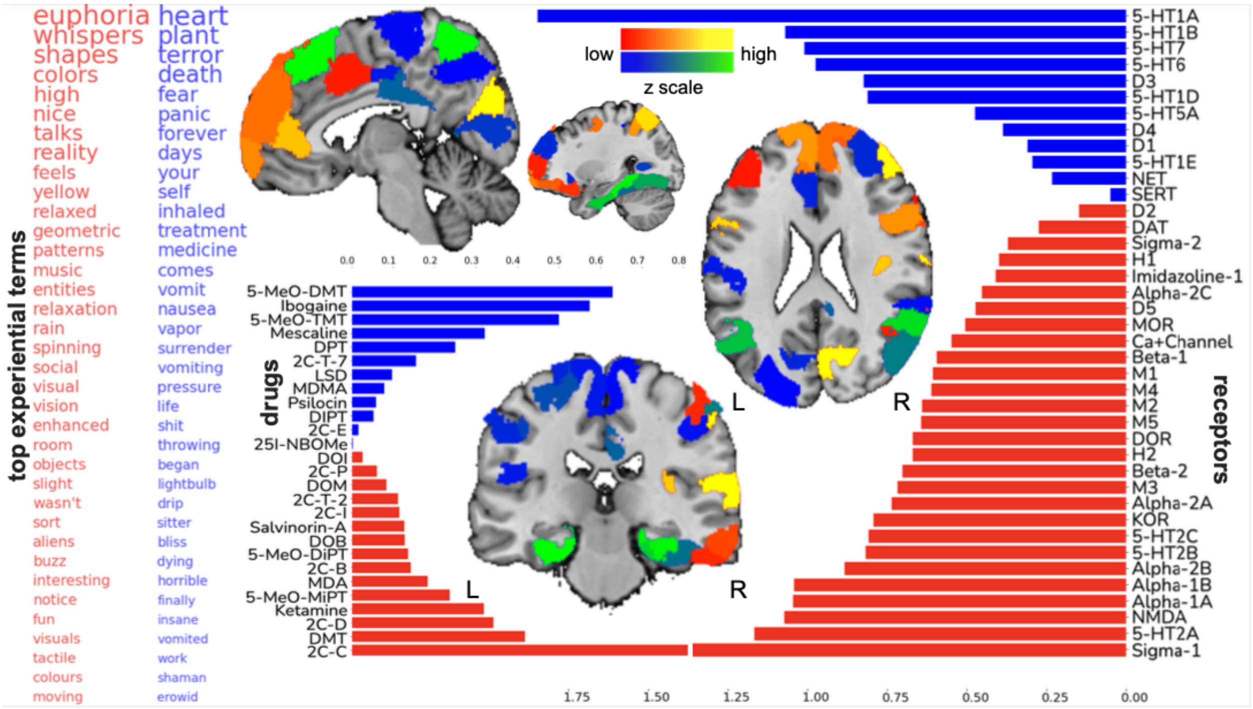

Supplementary Figure 3: The sixth factor underlying hallucinogenic experiences. Sagittal, coronal, and axial brain slices are shown at x=5, 28, y=-26, and z=23 (MNI reference space).

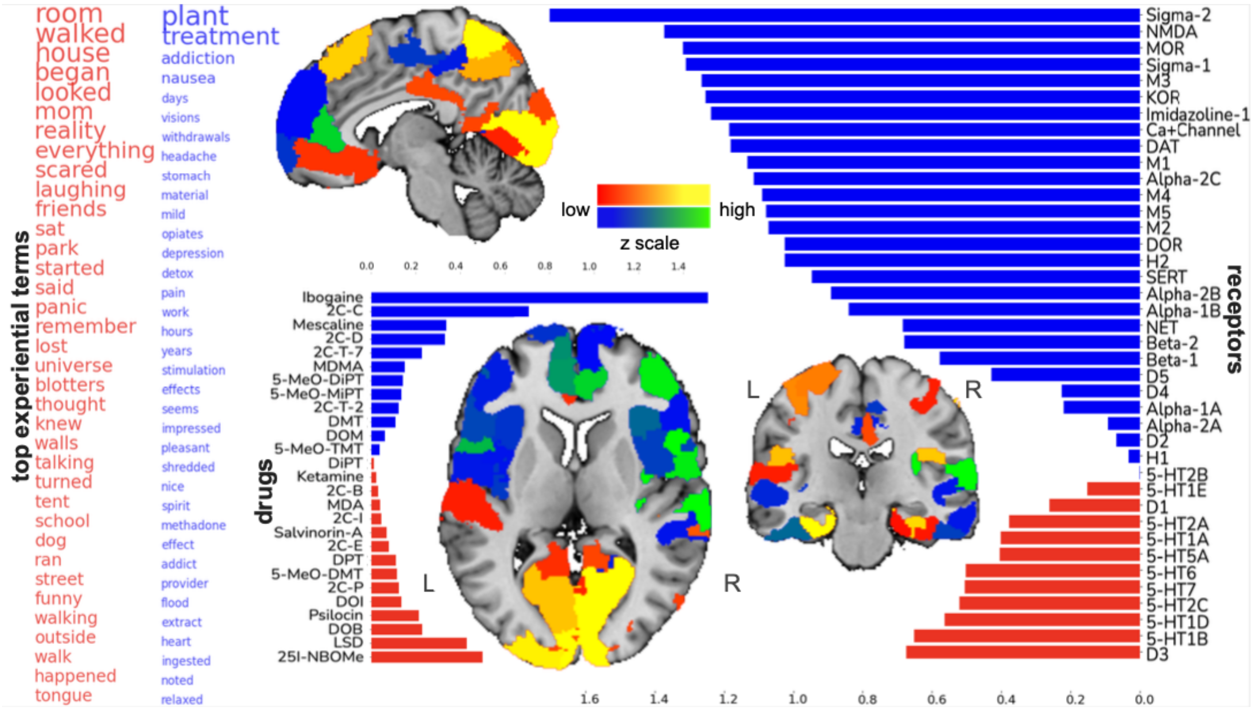

Supplementary Figure 4: The seventh factor underlying hallucinogenic experiences. Sagittal, coronal, and axial brain slices are shown at x=7, y=-23, and z=7 (MNI reference space).

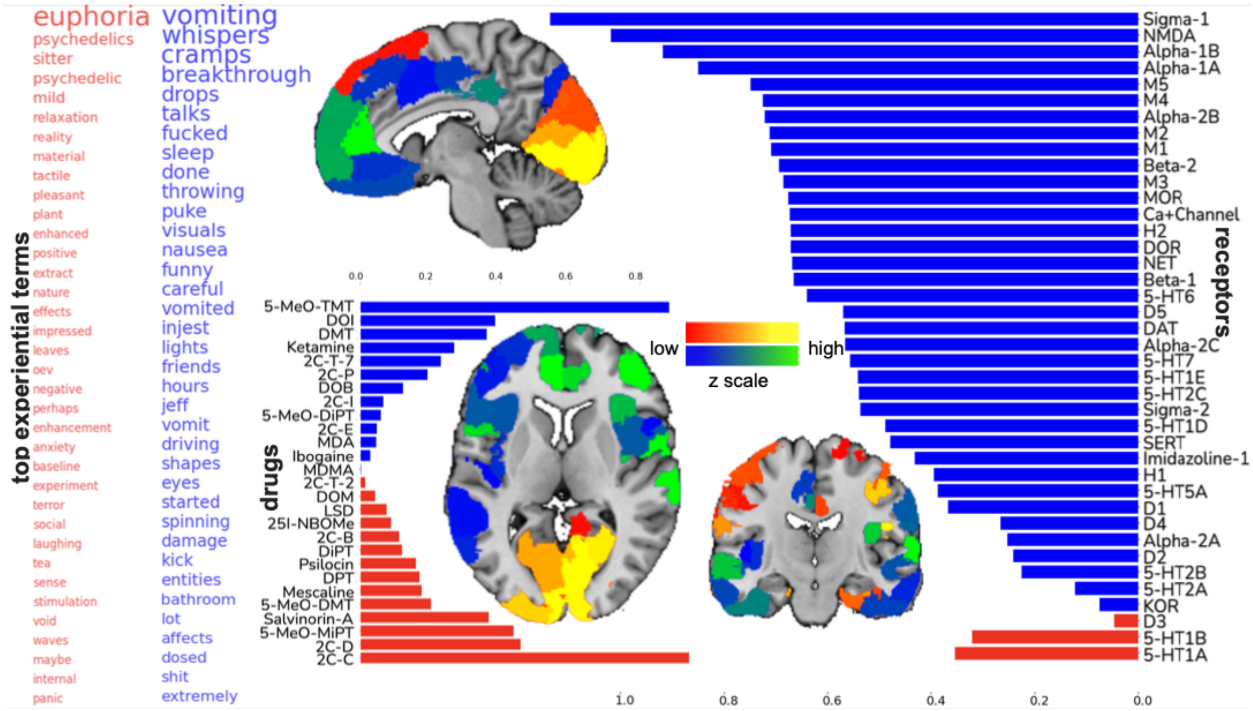

Supplementary Figure 5: The eighth factor underlying hallucinogenic experiences. Sagittal, coronal, and axial brain slices are shown at x=-6, y=-17, and z=6 (MNI reference space).

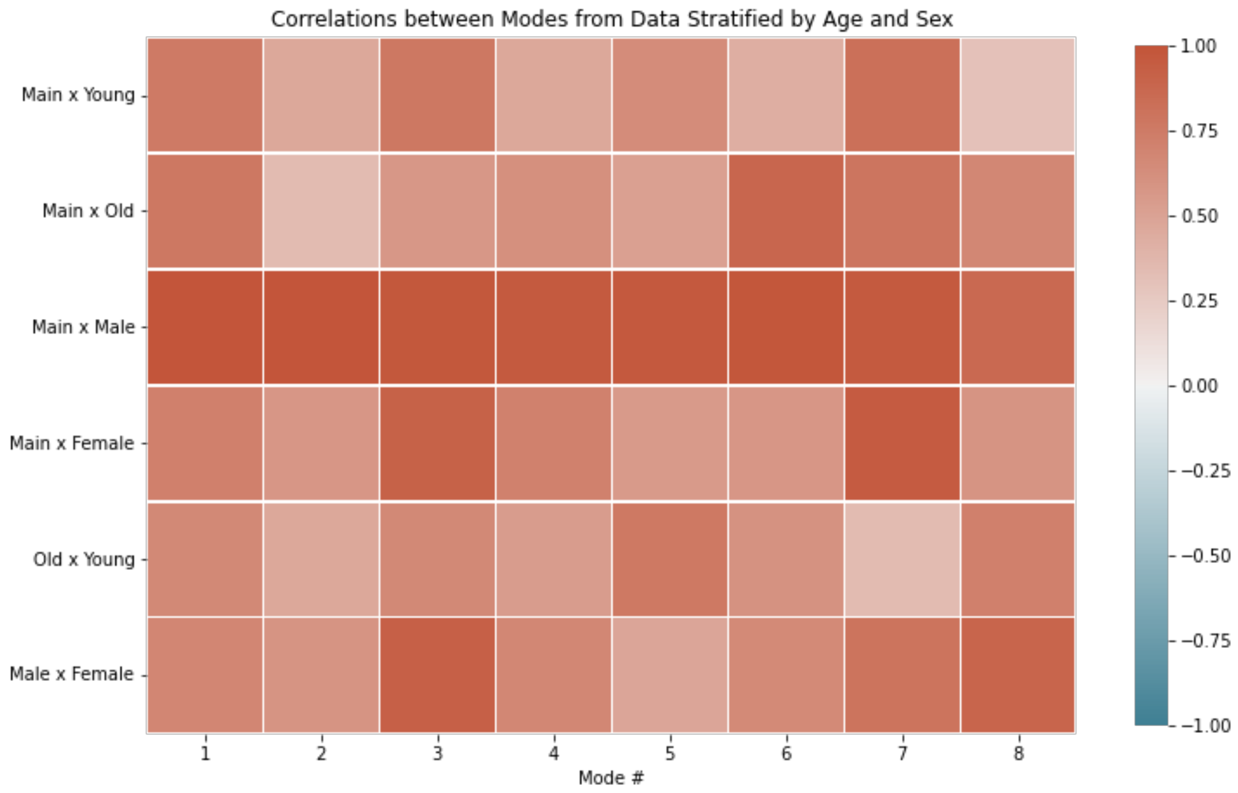

Supplementary Figure 6: Subselecting experience reports by age and sex shows agreement in modeling solutions. We have examined the possibility that the demographic characteristics age and sex may have unduly influenced our modeling results. To this end, we have constructed demographic subgroups by selecting only reports from older or younger individuals, or only from male or female individuals. For each of these subgroups, we have repeated the entire modeling pipeline, which yielded a set of receptor-experience factors for each subgroup. We then computed the similarity of these subgroup-specific modeling solutions based on Pearson’s correlation between the estimated model parameters (i.e., elements of the model-derived canonical vectors) obtained from two different demographic subgroups. These supplementary findings confirm that, focusing on certain age or sex strata, our results and scientific conclusions remain virtually identical.

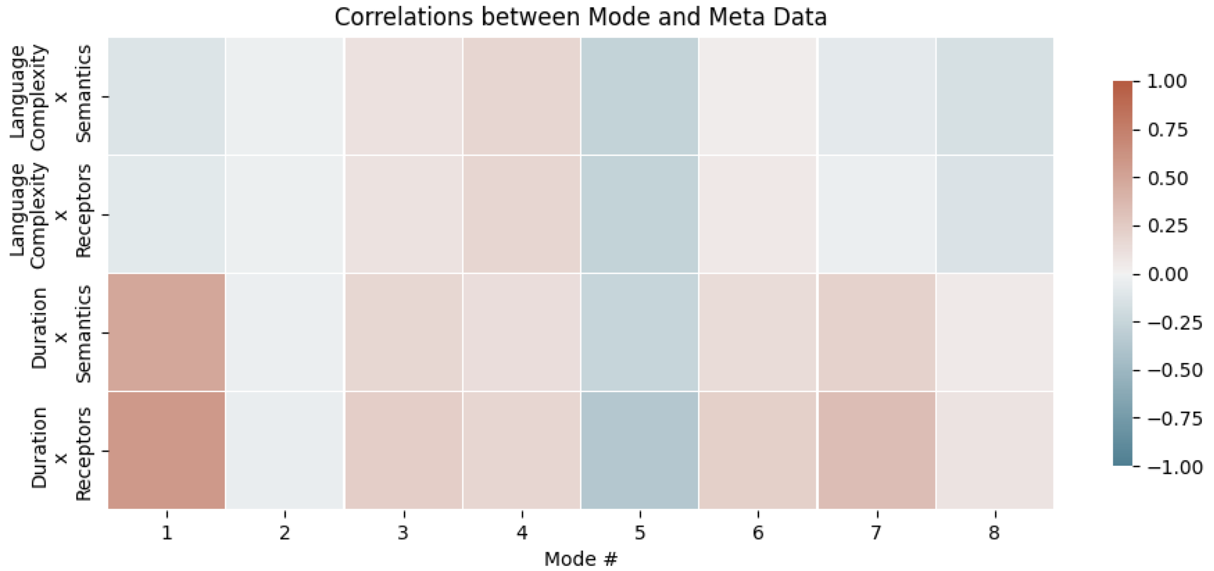

Supplementary Figure 7: The identified receptor-experience factors show only mild links to language complexity or duration of psychedelic experience. We have examined the possibility that the semantic structure of the experience reports or the length of drug experience may have unduly influenced our modeling results. To this end, language complexity was measured based on the number of distinct words that have been used in each experience report, as a proxy for the richness of employed verbal descriptions. The duration of experience was captured based on the usual length of time that a particular drug is known to show effects. We then quantified the association (Pearson's correlation rho) between these indicators and the expression strength of each receptor-experience factor (i.e., model-derived canonical variates).

Supplementary Table 2:
Receptor abbreviations

HTR1A,5HT1A
HTR2A,5HT2A
HTR1B,5HT1B
HTR1D,5HT1D
HTR1E,5HT1E
HTR2B,5HT2B
HTR2C,5HT2C
HTR5A,5HT5A
HTR6,5HT6
HTR7,5HT7
DRD1,D1
DRD2,D2
DRD3,D3
DRD4,D4
DRD5,D5
ADRA1A,Alpha1A
ADRA2A,Alpha2A
ADRA1B,Alpha1B
ADRA2B,Alpha2B
ADRA2C,Alpha2C
ADRB1,Beta1
ADRB2,Beta2
SLC6A4,SERT
SLC6A3,DAT
SLC6A2,NET
NISCH,Imidazoline1
SIGMAR1,Sigma1
TMEM97,Sigma2
OPRD1,DOR
OPRK1,KOR
OPRM1,MOR
CHRM1,M1
CHRM2,M2
CHRM3,M3
CHRM4,M4
CHRM5,M5
HRH1,H1
HRH2,H2
CACNA1C,Ca+Channel
GRIN1,NMDA
