# Supplementary material for: Trips and neurotransmitters: Discovering principled patterns across 6850 hallucinogenic experiences
Source: Sci Adv. 2022 Mar 16;8(11):eabl6989. doi: 10.1126/sciadv.abl6989 (PMC8926331; doi:10.1126/sciadv.abl6989)
Supplement: Supplementary file 2 — Table S1 [file sciadv.abl6989_table_s1.zip › sciadv.abl6989_table_s1.pdf]

Supplementary_Table_1

	5ht2a	5ht2c	5ht2b	5ht1a	5ht1b	5ht1d	5ht1e	5ht5a	5ht6	5ht7	D1	D2	D3	D4	D5	Alpha1A	Alpha1B
**25i-nbome**	1.666479593599830	4.60044666393478	>10000.0	1800.1139604180100	>10000.0	>10000.0	>10000.0	>10000.0	>10000.0	>10000.0	6700.388738077560	899.9119108700520	2099.9067037624300	>10000.0	>10000.0	369.9985311728600	>10000.0
**2c-b**	27.59942282854260	89.4952529765287	13.498950350559800	240.8241299827830	104.39988012574900	26.302679918953800	119.50883147362700	>10000.0	319.88951096914000	209.50770523651800	>10000.0	3371.3201787284300	7115.411150074330	>10000.0	>10000.0	>10000.0	>10000.0
**2c-c**	12.998702373886000	89.99119108700520	>10000.0	190.02030025772300	>10000.0	>10000.0	>10000.0	>10000.0	>10000.0	>10000.0	>10000.0	2099.9067037624300	>10000.0	>10000.0	>10000.0	>10000.0	>10000.0
**2c-d**	31.9963176731835	150.00301904559500	>10000.0	440.04794783598400	>10000.0	>10000.0	>10000.0	>10000.0	>10000.0	>10000.0	2399.937876809880	7100.680962545330	>10000.0	>10000.0	>10000.0	>10000.0	>10000.0
**2c-e**	43.903586564094500	104.11181076233400	25.1015188099533	307.3264929204290	252.8715671105160	73.1981323199808	626.3253647582230	>10000.0	2970.9818591187300	425.59841313374300	>10000.0	3338.8737672512300	1344.9309876881200	>10000.0	>10000.0	>10000.0	>10000.0
**2c-i**	3.5002575415843400	40.0036851046125	>10000.0	180.0113960418010	>10000.0	>10000.0	>10000.0	>10000.0	>10000.0	>10000.0	>10000.0	2699.6036086150100	5000.345349769780	>10000.0	>10000.0	5100.350239594460	>10000.0
**2c-p**	8.100279416803510	40.0036851046125	>10000.0	110.00185275062200	>10000.0	>10000.0	>10000.0	>10000.0	>10000.0	>10000.0	8400.40065263769	2299.8525866862000	5199.959965335160	>10000.0	>10000.0	3499.451670283570	>10000.0
**2c-t-2**	39.902490236214200	53.90139436325510	6.0006735386411600	381.24135110429900	857.4326166361800	85.90135215053960	415.14518004056900	>10000.0	1362.0717952990900	968.7238673827640	>10000.0	2795.1170539918500	1834.8476077555400	>10000.0	>10000.0	>10000.0	>10000.0
**2c-t-7**	6.499800096830250	39.00317844750660	>10000.0	519.9959965335160	>10000.0	>10000.0	>10000.0	>10000.0	>10000.0	>10000.0	>10000.0	5000.345349769780	7500.6689873606900	>10000.0	>10000.0	>10000.0	>10000.0
**5-meo-dipt**	>10000.0	>10000.0	163.0046523984840	132.40366293918900	5136.886854227250	1717.9083871575900	>10000.0	>10000.0	>10000.0	1231.1189039637800	>10000.0	>10000.0	>10000.0	>10000.0	>10000.0	>10000.0	>10000.0
**5-meo-dmt**	2010.9441078951400	538.0219571891120	3884.1858339296300	1.9002030025772300	73.9945954312724	6.299411805485590	360.1637503947280	276.6304606897500	35.497682446720900	3.9003178447506600	79.506017508442	3562.049907308450	497.62248997346900	3120.326216598010	>10000.0	>10000.0	>10000.0
**5-meo-mipt**	448.3322704327330	2186.250893606700	58.505945195000600	12.299855235704700	302.83076942657800	22.50090572279310	3496.2300400270200	952.7961640236520	130.1967070064140	19.801587080824100	>10000.0	>10000.0	2470.017328046610	6331.403617108160	>10000.0	>10000.0	>10000.0
**5-meo-tmt**	>10000.0	4019.758849668720	>10000.0	199.9861869632750	>10000.0	249.97697021785100	1800.1139604180100	10449.608026382400	60.00673538641160	145.01068364219700	>10000.0	>10000.0	>10000.0	>10000.0	>10000.0	>10000.0	>10000.0
**dipt**	>10000.0	>10000.0	398.4740097177750	120.50359403718000	>10000.0	3741.9674033666400	>10000.0	>10000.0	>10000.0	3422.9456231604100	>10000.0	>10000.0	3321.2380132514600	>10000.0	>10000.0	>10000.0	>10000.0
**dmt**	2322.7367963571100	334.58001785330100	107.59695972076900	>10000.0	>10000.0	93.00365147539600	455.72200558864700	610.9420249055720	487.4162454431540	87.4983775227436	271.0815748427230	>10000.0	>10000.0	>10000.0	>10000.0	1745.0183572026500	973.6435697988860
**dob**	23.099362801731900	41.495404263436300	3.9003178447506600	2549.7640877992800	940.8058300365890	635.477239048616	1426.9212315793600	5311.289818550130	5534.775374571610	506.290758346022	>10000.0	>10000.0	808.1649291125380	>10000.0	>10000.0	>10000.0	>10000.0
**doi**	165.3864784771330	45.80364077908270	335.89226273449500	2219.218172251270	>10000.0	457.82552279169400	1012.9779687601000	>10000.0	2113.002447024130	5768.992840692840	9687.238673827640	>10000.0	>10000.0	>10000.0	>10000.0	>10000.0	>10000.0
**dom**	507.45787908065000	3980.155135426720	11.700380894230500	3655.9479161312500	>10000.0	208.88151080233900	3541.6040042646200	>10000.0	8154.550000611170	1591.1092480175400	>10000.0	>10000.0	>10000.0	>10000.0	>10000.0	3218.844524111850	>10000.0
**dpt**	2579.2889644274900	1567.1118694109600	41.995233466406600	31.79801427691530	854.476257684183	618.8708114190300	2337.7604122746900	4373.2066516964500	4542.552967388710	283.92262394249100	>10000.0	9249.111180764650	1361.1312343313100	2014.1879803932000	>10000.0	880.8460273622600	443.404401633441
**ibogaine**	14141.647250701500	>10000.0	>10000.0	>10000.0	>10000.0	>10000.0	>10000.0	>10000.0	>10000.0	>10000.0	>10000.0	>10000.0	70000.31591308980	>10000.0	>10000.0	>10000.0	>10000.0
**ketamine**	>10000.0	>10000.0	>10000.0	>10000.0	>10000.0	>10000.0	>10000.0	>10000.0	>10000.0	>10000.0	>10000.0	>10000.0	>10000.0	>10000.0	>10000.0	>10000.0	>10000.0
**lsd**	11.300560897485400	30.598490346550000	29.998531811907900	7.299615757292980	3.9003178447506600	7.800096937165000	92.89663867799360	8.999119108700520	6.899220098440470	6.599332342635640	177.0108958317420	110.10321487297700	27.00225286888240	158.4163490206370	344.4292293943150	1127.7166693314700	8677.607449911470
**mda**	>10000.0	6418.0042448391800	90.99132726322520	3761.8372279132800	>10000.0	>10000.0	>10000.0	>10000.0	>10000.0	3547.316998366910	>10000.0	>10000.0	>10000.0	>10000.0	>10000.0	>10000.0	>10000.0
**mdma**	>10000.0	>10000.0	500.0345349769780	>10000.0	>10000.0	>10000.0	>10000.0	>10000.0	>10000.0	>10000.0	>10000.0	>10000.0	>10000.0	>10000.0	>10000.0	>10000.0	>10000.0
**mescaline**	>10000.0	>10000.0	792.8663748785480	1841.1959036876500	>10000.0	>10000.0	5204.751511705230	>10000.0	>10000.0	>10000.0	>10000.0	>10000.0	>10000.0	>10000.0	>10000.0	>10000.0	>10000.0
**psilocin**	339.62527259040900	141.18871968036600	4.70002318179801	62.60369976726430	305.0001128249180	18.599445632225800	44.299619910036400	69.90367367619770	71.69683755804200	71.59785310819620	19.90215022279340	>10000.0	101.20454110965400	>10000.0	>10000.0	>10000.0	>10000.0
**salvinorin-a**	>10000.0	>10000.0	>10000.0	>10000.0	>10000.0	>10000.0	>10000.0	>10000.0	>10000.0	>10000.0	>10000.0	>10000.0	>10000.0	>10000.0	>10000.0	>10000.0	>10000.0

Alpha2A	Alpha2B	Alpha2C	Beta1	Beta2	SERT	DAT	NET	Imidazoline1	Sigma1	Sigma2	DOR	KOR	MOR	M1	M2	M3	M4
319.963176731835	>10000.0	>10000.0	>10000.0	>10000.0	1000.0	5400.077590665930	1299.8702373886000	>10000.0	>10000.0	>10000.0	>10000.0	>10000.0	>10000.0	>10000.0	>10000.0	>10000.0	>10000.0
308.8161469184510	664.8137604740010	103.29992352432300	>10000.0	>10000.0	>10000.0	>10000.0	>10000.0	2155.261643566160	>10000.0	>10000.0	>10000.0	>10000.0	>10000.0	>10000.0	>10000.0	821.6748609764340	>10000.0
530.0294488357960	>10000.0	>10000.0	>10000.0	>10000.0	>10000.0	>10000.0	>10000.0	>10000.0	>10000.0	>10000.0	>10000.0	>10000.0	>10000.0	>10000.0	>10000.0	>10000.0	>10000.0
100.0	>10000.0	>10000.0	>10000.0	>10000.0	>10000.0	>10000.0	>10000.0	>10000.0	>10000.0	>10000.0	>10000.0	>10000.0	>10000.0	>10000.0	>10000.0	>10000.0	>10000.0
490.1172634719430	305.49211132155100	90.19864220737450	>10000.0	>10000.0	>10000.0	>10000.0	>10000.0	>10000.0	>10000.0	>10000.0	>10000.0	>10000.0	>10000.0	>10000.0	>10000.0	2556.2304271125500	>10000.0
70.00031591308970	>10000.0	>10000.0	>10000.0	>10000.0	4900.044227932360	>10000.0	>10000.0	>10000.0	>10000.0	>10000.0	>10000.0	>10000.0	>10000.0	>10000.0	>10000.0	>10000.0	>10000.0
89.99119108700520	>10000.0	>10000.0	>10000.0	>10000.0	>10000.0	>10000.0	>10000.0	>10000.0	>10000.0	>10000.0	>10000.0	>10000.0	>10000.0	>10000.0	>10000.0	>10000.0	>10000.0
729.6254933925090	981.7479430199840	165.88228153750500	9202.37654805671	1184.1316792087600	>10000.0	>10000.0	>10000.0	2080.1756100985900	3869.9022919026900	>10000.0	>10000.0	>10000.0	>10000.0	>10000.0	>10000.0	692.149644226647	>10000.0
180.0113960418010	>10000.0	>10000.0	>10000.0	>10000.0	>10000.0	>10000.0	>10000.0	>10000.0	>10000.0	>10000.0	>10000.0	>10000.0	>10000.0	>10000.0	>10000.0	>10000.0	>10000.0
>10000.0	5292.97691687475	2864.8375471900300	>10000.0	>10000.0	2531.0457771974400	>10000.0	>10000.0	759.4514226678700	9442.782793644790	3001.9261222416900	>10000.0	>10000.0	>10000.0	>10000.0	>10000.0	>10000.0	>10000.0
1890.1662311869000	2639.9761293548100	508.0424479948440	>10000.0	>10000.0	2031.8890973131300	>10000.0	2858.9068155119600	2498.04351871415	>10000.0	>10000.0	>10000.0	>10000.0	>10000.0	>10000.0	>10000.0	>10000.0	>10000.0
174.98466886246600	1693.1677975100100	636.9421653777090	>10000.0	>10000.0	6409.143566447760	>10000.0	>10000.0	879.4274148836830	>10000.0	917.9097860573370	>10000.0	>10000.0	>10000.0	>10000.0	>10000.0	>10000.0	>10000.0
>10000.0	>10000.0	>10000.0	>10000.0	>10000.0	>10000.0	>10000.0	6379.696226625790	>10000.0	>10000.0	>10000.0	>10000.0	>10000.0	>10000.0	>10000.0	>10000.0	>10000.0	>10000.0
>10000.0	2870.119636464440	2522.899786450710	>10000.0	>10000.0	1258.0560772203400	>10000.0	>10000.0	355.959018006083	1798.0426951774400	2702.091180818970	>10000.0	>10000.0	>10000.0	>10000.0	>10000.0	>10000.0	>10000.0
1560.9895654550600	257.69144451738700	258.58301804210600	>10000.0	>10000.0	3741.9674033666400	>10000.0	>10000.0	650.2794054723700	5209.547473285550	>10000.0	>10000.0	>10000.0	>10000.0	>10000.0	>10000.0	>10000.0	>10000.0
4265.795188015930	1526.8627500263500	593.7450473021950	2424.9344310334700	302.9005068166220	8538.862102148430	>10000.0	>10000.0	1596.2466542993900	2192.80493535045	>10000.0	>10000.0	>10000.0	>10000.0	>10000.0	>10000.0	1151.5956083180300	>10000.0
73.70551753379340	340.01650580407100	600.6202909194720	590.8809655747820	138.61173512382400	684.6994830911340	>10000.0	>10000.0	>10000.0	8564.460288900580	9172.759353897790	>10000.0	>10000.0	>10000.0	2720.1947619843800	1988.8407051736500	1427.9072543673800	577.6968506969280
579.9626158196420	873.7757751180200	921.2977276685560	>10000.0	48.89900250464930	>10000.0	>10000.0	>10000.0	>10000.0	>10000.0	>10000.0	>10000.0	>10000.0	>10000.0	>10000.0	>10000.0	>10000.0	>10000.0
457.61473488633300	339.31261016293200	514.3988630287730	>10000.0	>10000.0	156.71118694109600	1500.0301904559500	>10000.0	340.01650580407100	396.8258909509110	2916.755328939500	>10000.0	>10000.0	>10000.0	>10000.0	>10000.0	>10000.0	>10000.0
>10000.0	>10000.0	>10000.0	>10000.0	>10000.0	548.6558320717440	1980.1587080824100	>10000.0	>10000.0	5838.482731072000	206.0155490320010	>10000.0	2717.064824112680	4362.144019080490	22485.368014916200	39409.41607322860	12499.711805783700	>10000.0
>10000.0	>10000.0	>10000.0	>10000.0	>10000.0	>10000.0	>10000.0	>10000.0	>10000.0	945.999999996095	>10000.0	>10000.0	>10000.0	>10000.0	>10000.0	>10000.0	>10000.0	>10000.0
45.80364077908270	>10000.0	>10000.0	1600.6633458753900	3460.9872548535300	>10000.0	>10000.0	>10000.0	>10000.0	>10000.0	>10000.0	>10000.0	>10000.0	>10000.0	>10000.0	>10000.0	>10000.0	>10000.0
1655.0076287699900	690.239803840242	228.9812912107970	>10000.0	>10000.0	>10000.0	>10000.0	>10000.0	>10000.0	>10000.0	>10000.0	>10000.0	>10000.0	>10000.0	>10000.0	>10000.0	>10000.0	>10000.0
2532.211635280830	1784.8429164980100	1345.8603540559500	>10000.0	>10000.0	>10000.0	>10000.0	>10000.0	219.68479539297200	>10000.0	>10000.0	>10000.0	>10000.0	>10000.0	>10000.0	>10000.0	1850.9726429316500	8245.177320439840
8930.998162235170	>10000.0	745.2465942515140	>10000.0	>10000.0	>10000.0	>10000.0	>10000.0	2677.3182599684600	>10000.0	>10000.0	>10000.0	>10000.0	>10000.0	>10000.0	>10000.0	>10000.0	>10000.0
2044.089936002380	1270.8666993928200	4403.520272026520	>10000.0	>10000.0	851.5300920209450	>10000.0	>10000.0	792.1364541528510	>10000.0	>10000.0	>10000.0	>10000.0	>10000.0	>10000.0	>10000.0	>10000.0	>10000.0
>10000.0	>10000.0	>10000.0	>10000.0	>10000.0	>10000.0	>10000.0	>10000.0	>10000.0	>10000.0	>10000.0	>10000.0	4.300312336305440	>10000.0	>10000.0	>10000.0	>10000.0	>10000.0

M5	H1	H2	Ca+Channel	NMDA	Source
>10000.0	89.99119108700520	>10000.0	>10000.0	>10000.0	(23)
>10000.0	>10000.0	>10000.0	>10000.0	>10000.0	(15)
>10000.0	>10000.0	>10000.0	>10000.0	>10000.0	(23)
>10000.0	>10000.0	>10000.0	>10000.0	>10000.0	(23)
1725.043297245840	>10000.0	>10000.0	>10000.0	>10000.0	(15)
>10000.0	6099.580958722390	>10000.0	>10000.0	>10000.0	(23)
>10000.0	>10000.0	>10000.0	>10000.0	>10000.0	(23)
1502.1039909500800	>10000.0	>10000.0	>10000.0	>10000.0	(15)
>10000.0	>10000.0	>10000.0	>10000.0	>10000.0	(23)
>10000.0	>10000.0	>10000.0	>10000.0	>10000.0	(15)
>10000.0	>10000.0	>10000.0	>10000.0	>10000.0	(15)
>10000.0	4819.477976251270	>10000.0	>10000.0	>10000.0	(15)
>10000.0	>10000.0	>10000.0	>10000.0	>10000.0	(15)
>10000.0	3582.613845838670	>10000.0	>10000.0	>10000.0	(15)
>10000.0	>10000.0	>10000.0	>10000.0	>10000.0	(15)
>10000.0	9120.108393559100	>10000.0	>10000.0	>10000.0	(15)
2208.004733018900	1757.114246595100	>10000.0	>10000.0	>10000.0	(15)
>10000.0	>10000.0	>10000.0	>10000.0	>10000.0	(15)
>10000.0	125.11229769215900	>10000.0	>10000.0	>10000.0	(15)
>10000.0	>10000.0	>10000.0	>10000.0	2000.783052152390	(15)
>10000.0	>10000.0	>10000.0	>10000.0	478.99999999496700	(15)
>10000.0	1543.4764233245300	>10000.0	>10000.0	>10000.0	(15)
>10000.0	>10000.0	>10000.0	>10000.0	>10000.0	(15)
6329.9459253789200	>10000.0	>10000.0	1198.119123190030	>10000.0	(15)
>10000.0	>10000.0	>10000.0	>10000.0	>10000.0	(15)
>10000.0	>10000.0	>10000.0	>10000.0	>10000.0	(15)
>10000.0	>10000.0	>10000.0	>10000.0	>10000.0	(15)
